# Electrospun Cellulose-Acetate/Chitosan Fibers for Humic-Acid Removal: Improved Efficiency and Robustness with a Core-Sheath Design

**DOI:** 10.3390/nano12081284

**Published:** 2022-04-09

**Authors:** Yirong Zhang, Yixiang Wang

**Affiliations:** Department of Food Science and Agricultural Chemistry, McGill University, Ste Anne de Bellevue, QC H9X 3V9, Canada; yirong.zhang@mail.mcgill.ca

**Keywords:** co-axial electrospinning, cellulose nanocrystals, cellulose acetate, chitosan, adsorbents

## Abstract

Recycling biomass waste into functional materials has attracted much attention, and a rational structural design can make more effective use of each component. In our previous work, the fabrication of electrospun cellulose-acetate (CA)/chitosan (CS) adsorbents for humic-acid (HA) removal guided by the intermolecular interaction mechanism was demonstrated. Herein, a core-sheath structure was designed via one-step co-axial electrospinning, where a mixture of CS and CA was employed as the sheath layer to efficiently adsorb HA, and cellulose nanocrystals (CNCs) derived from waste cotton fabrics were incorporated into the CA core as load-bearing components. Compared to the non-layered electrospun CS/CA fibers, all the CS/CA–CNC fibers with a core-sheath structure exhibited smaller diameters, greater homogeneity, and significantly improved mechanical strength. Meanwhile, their maximum adsorption capacities towards HA had no significant differences. Even after the complete hydrolysis of CA into cellulose, the electrospun fibers maintained the fibrous structures and showed a higher tensile strength while exhibiting an acceptable adsorption capacity towards HA. Therefore, this work demonstrates the importance of rational design in the efficient preparation of functional materials and the feasibility of using electrospun core-sheath fibers derived from biomass wastes for the removal of water contaminants.

## 1. Introduction

The fair utilization and recycling of biomass waste is an important consideration of the circular economy where as much value as possible is retained towards zero waste [1]. Nevertheless, a great amount of biomass wastes from agriculture, forestry, and fisheries have instead been annually processed by combustion and landfilling, leading to a series of environmental problems [2,3,4,5]. Forestry biomass was reported to be the largest source of biomass input in Canada and reached 12.3 million metric tons in 2015 [6]. Among the wood-derived compounds, cellulose acetate (CA) is recognized as one of the most important derivatives in terms of its considerable commercial contribution [7]. CA has been extensively used to produce consumer products comprising cigarette filters, textile fibers, films, plastics, and others [8], the majority of which have no sustainable disposal methods. Additionally, industrial applications of chitosan (CS) and cellulose in the agriculture, pharmaceutical, and textile sectors are exponentially expanding to meet the market demand, which will continuously generate wastes in the foreseeable future [9]. Recycling and converting these biomass wastes and residues into value-added products is a wise strategy to relieve pressure on the environment.

Recently, electrospun fibrous membranes have drawn extensive attention and demonstrated promising applications in the fields of tissue engineering, filtration, and sensing, owing to their high porosity, large specific area, simple operation, and good interconnectivity [10,11,12,13,14]. Among them, electrospun CA materials have specifically shown great potential in water treatment [11,15]. In our previous work, the feasibility of using an intermolecular-interaction study to guide the fabrication of electrospun CA/CS adsorbents for efficient humic-acid (HA) removal was demonstrated, and the superior adsorption performance toward HA was due to the electrostatic and hydrophobic interactions [16]. It is worth noting that adsorption mainly relies on the active sites on the surface of adsorbents for the uptake of contaminants [17], and the mechanical strength of the adsorbent plays a vital role in commercial applications because it guarantees the integrity of the electrospun fibrous membranes during water treatment [10]. Therefore, to further improve the performance and mechanical properties of the adsorbents, rationally designed structures of the fibrous composites are extremely important, which can also contribute to cost savings in water treatment by using a minimum amount of functional components [18]. Co-axial electrospinning is a double-fluid process whereby two individual solutions are simultaneously spun through the co-axial capillaries to obtain nanofibers with core-sheath structures [19,20]. The unique features endow co-axial electrospinning with several advantages over the conventional configuration in terms of flexibility and selection of polymers/solvents when designing and constructing functional materials [21,22,23]. Given these advantages, co-axial electrospinning has been used to prepare adsorbents with excellent removal effects. For example, the core-sheath structure of CA (core)–hydroxyapatite (sheath) fibers was developed for adsorbing bovine serum albumin, which reached a maximum adsorption capacity of 176.04 mg/g [19].

In this study, CA from cigarette filters, CS from crab shells, and cellulose nanocrystals (CNCs) from waste cotton textiles were selected to fabricate the core-sheath-structured adsorbents via one-step co-axial electrospinning. Specifically, a mixture of CS and CA was employed as the sheath layer to efficiently adsorb HA, and CNCs were incorporated in the CA core as load-bearing components. The effect of the CNC contents on the structure and mechanical properties of the core-sheath CS/CA–CNC fibers was studied via scanning electron microscopy (SEM), Fourier-transform infrared (FTIR) spectroscopy, and the uniaxial tensile test. Additionally, the adsorption performance of the core-sheath CS/CA–CNC fibers and the hydrolyzed fibers was investigated and compared.

## 2. Materials and Methods

### 2.1. Materials

CA from cigarette filters was kindly provided by Celanese Corporation (Irving, TX, USA. Mw = 75–95 kDa, acetyl content of 39.95 wt%, degree of substitution of ~2.47). CS derived from crab shells was kindly provided by Dr. Benjamin K. Simpson (Department of Food Science and Agricultural Chemistry, McGill University, Quebec, Canada. Molecular weight 190–310 kDa, degree of deacetylation 75–85%). CNCs with lengths and diameters of 111.76 ± 38.73 nm and 11.18 ± 2.33 nm were extracted from waste cotton fabrics via sulfuric-acid hydrolysis [24]. Acetic acid (CH_3_COOH, 100%), HA (sodium salt, C_9_H_8_Na_2_O_4_ 45–70%), sodium hydroxide (NaOH, ACS reagent grade), and sulfuric acid (H_2_SO_4_, ACS reagent grade) were purchased from Fisher Scientific (Mississauga, ON, Canada). All the reagents were used as received without further purification. Deionized water was used to prepare the electrospinning solutions and HA solutions.

### 2.2. Core-Sheath CS/CA–CNC-Fiber Fabrication

For the co-axial-electrospinning configuration, the CS/CA ratios of 1:1 *w*/*w* and 3:1 *w*/*w* that were optimized in our previous work were chosen to make the sheath layer [16], and the core layer was prepared by adding CNCs (3 wt% and 5 wt% of CA dry weight) to a 12 wt% CA solution. A summary of sample compositions and electrospinning conditions is listed in Table 1. The core and sheath fluids were loaded into two syringes and forced through stainless-steel needles with concentric structures. The diameters of the inner and outer needles were 0.66 mm and 1.57 mm, respectively. A stainless-steel drum rotating at 10 rpm was used to collect the fibers. The obtained fibrous membranes were vacuum dried in a desiccator at room temperature for 24 h to remove the solvent residue. In order to investigate the effect of deacetylation, 1:1CS/CA–5%CNCs and 3:1CS/CA–5%CNCs were immersed in 20 mL of 0.5 M NaOH/ EtOH solution and stirred for 1 h at room temperature. The membranes were then washed with distilled water several times until the pH level of the wastewater was neutral, and vacuum dried in a desiccator at room temperature for 24 h. The deacetylated fibers were coded as 1:1CS/CL–5%CNCs and 3:1CS/CL–5%CNCs.

### 2.3. Core-Sheath CS/CA–CNC-Fiber Characterization

Morphological characterization of the core-sheath CS/CA–CNC fibers was carried out with a Hitachi SU-3500 SEM (Hitachi, Tokyo, Japan) operating at 30 kV. Prior to the SEM observation, the samples were coated with 4 nm of gold/platinum coating using a Leica EM ACE200 coater (Leica, Wetzlar, Germany). Fiber diameters were measured with the ImageJ image-visualization software developed by the National Institute of Health. Specifically, SEM images under a magnification of ×10 k were selected, and for each sample, six hundred random positions were measured [25]. FTIR spectra of the core-sheath CS/CA–CNC fibers were recorded on a Varian Excalibur 3100 FTIR spectrometer (Varian, Melbourne, Australia) equipped with an attenuated total-reflectance accessory (Specac, Orpington, UK) as the average of 64 scans with a resolution of 4 cm^–1^. The uniaxial tensile test of the CS/CA–CNC fibers was performed to evaluate their tensile strength and elongation at break. Seven specimens with dimensions of 30 mm × 10 mm (length × width) were tested for each sample using an ADMET eXpert 7601 Test System (ADMET, Norwood, MA, USA) at a fixed crosshead velocity of 1 mm·min^−1^. The initial grip-separation distance was fixed at 10 mm. The thickness of each specimen was measured using the ImageJ image-visualization software. The tensile strength (*σ*) of CS/CA fibrous films was calculated using the following equation:

(1) $$\sigma =\frac{F}{A}\;$$

where *F* (N) is the maximum load at break, and *A* (mm^2^) is the cross-sectional area.

### 2.4. Adsorption Experiment

Adsorption of HA was determined as functions of CS/CA ratios in the sheath, CNCs contents in the core, and adsorption time under the following optimized conditions (pH: 4.0, adsorbent dosage: 1.2 mg, volume of HA solution: 20 mL, initial concentration of HA solution: 30 ppm, shaking speed: 155 rpm, and temperature: 25 °C) [16]. Stock solution with a concentration of 100 ppm HA was prepared and then diluted to obtain 30 ppm HA solution. Sulfuric acid was used to adjust the pH value of HA solution to pH 4.0. Batch experiment was carried out in 25 mL glass bottles with white polypropylene caps, and 1.2 mg of adsorbents was added and shaken for desirable time intervals at 155 rpm. The concentrations of HA in the solutions before and after the adsorption process were determined using a Hitachi UV-2000 UV–Vis spectrophotometer (Hitachi, Tokyo, Japan) at a wavelength of 278 nm. The adsorption capacity was calculated using the following equation:

(2) $$\;q_{e}\left(mg\cdot g^{-1}\right)=\frac{\left(C_{0}-C_{e}\right)\times V}{m}$$

where *C*_0_ (mg/L) is the initial HA concentration in the solution, *C_e_* (mg/L) is the equilibrium HA concentration, *V* (L) is the volume of HA solution, and *m* (g) is the mass of electrospun adsorbent.

### 2.5. Statistical Analysis

All experimental results were expressed as the mean of at least three replicas ± SD. Statistical interpretations of the experimental results were carried out by analysis of variance (ANOVA) followed by multiple comparison tests using Duncan’s multiple-range test at the 95% confidence level. All analyses were conducted using SPSS statistical software (version 27, IBM, Armonk, NY, USA) with a probability of *p* < 0.05 considered to be significant.

## 3. Results and Discussion

### 3.1. Core-Sheath CS/CA–CNC-Fiber Structure

**Figure 1:**
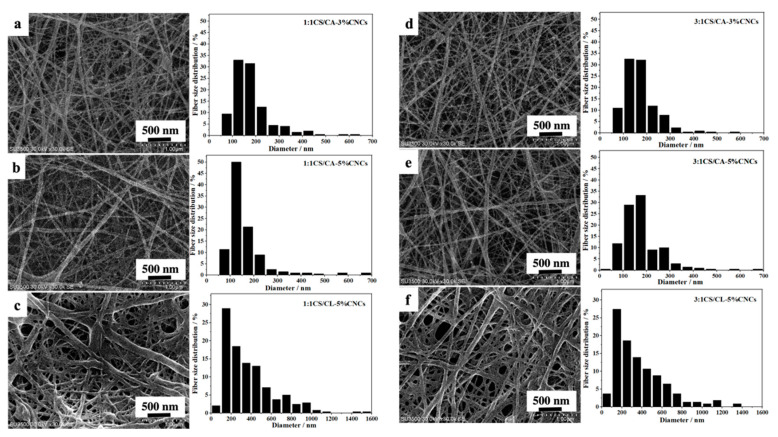
SEM images and fiber-diameter distributions of (**a**) 1:1CS/CA–3%CNCs, (**b**) 1:1CS/CA–5%CNCs, (**c**) 1:1CS/CL–5%CNCs, (**d**) 3:1CS/CA–3%CNCs, (**e**) 3:1CS/CA–5%CNCs, and (**f**) 3:1CS/CL–5%CNCs.

Figure 1 shows the morphology and corresponding diameter distribution of the core-sheath-structured fibers with various CNC contents. The average fiber diameters of 1:1CS/CA–5%CNCs, 1:1CS/CA–3%CNCs, 3:1CS/CA–5%CNCs, and 3:1CS/CA–3%CNCs samples were 174.7 ± 89.6, 158.8 ± 66.0, 172.4 ± 84.3, and 168.1 ± 63.2 nm, respectively. All the CS/CA–CNC adsorbents exhibited fine fibrous structures, which facilitated the adsorption of water contaminants [26,27]. The average fiber diameters of both 1:1CS/CA–CNC and 3:1CS/CA–CNC samples increased when the loading amount of CNCs was adjusted from 3 wt% to 5 wt%. It was because the viscosity of electrospun solutions increased perceptibly as the CNC content rose [28,29], and the higher viscosity induced stronger resistance to the stretching force generated axially by the electric field [10,30]. The high viscosity also caused the formation and deposition of a few convex- and concave-shaped fibers, as shown in Figure 1b, e. In addition, the variance in fiber diameters became greater as the loading level of CNCs increased, which was indicated by the large standard deviation and more spread-out distribution of the fiber diameters (Figure 1). In other words, the homogeneity of the 1:1CS/CA–3%CNCs and 3:1CS/CA–3%CNCs fibers were greater than that of the samples with 5 wt% of CNCs. Different amounts of CNCs could have an impact on the dispersible degree of CNCs in the core-layer solution. Hence, the wider distribution of fiber diameters could be explained by the unstable Taylor cone in the presence of a higher amount of CNCs. Similar phenomena were also observed and reported by Ni et al. [31] and Patiño Vidal et al. [32].

Compared to the non-layered CS/CA fibers reported in our previous work [16], all the fiber diameters of the core-sheath CS/CA–CNC fibers were smaller with greater uniformity in terms of diameter distributions. This observation could be associated with the poor electrospinnability of CS and a lower mass proportion of CS in the core-sheath fibers. After alkali hydrolysis, both 1:1CS/CA–5%CNCs and 3:1CS/CA–5%CNCs maintained their fiber integrity and porous structure. Nevertheless, the CS/CL–CNC fibers swelled in water due to the improved hydrophilicity.

**Figure 2:**
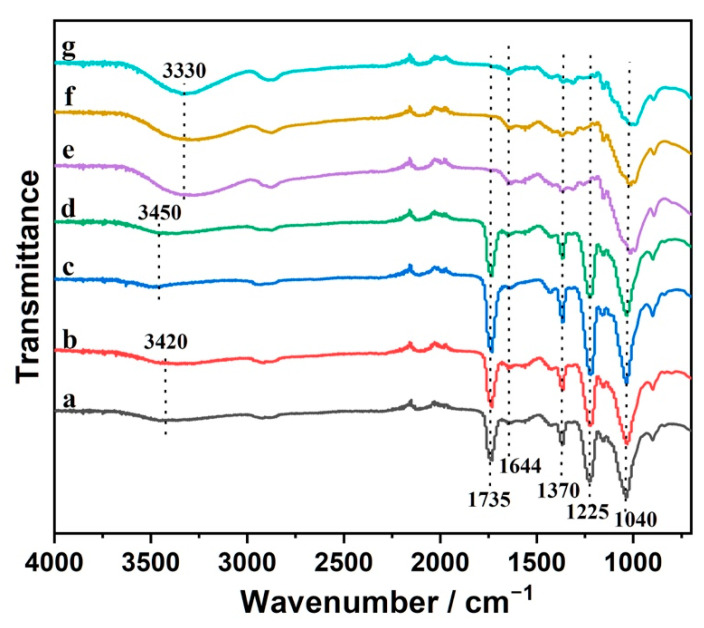
FTIR spectra of electrospun fibers: (**a**) 3:1CS/CA–3%CNCs, (**b**) 3:1CS/CA–5%CNCs, (**c**) 1:1CS/CA–3%CNCs, (**d**) 1:1CS/CA–5%CNCs, (**e**) 1:1CS/CL–5%CNCs, and (**f**) 3:1CS/CL–5%CNCs, and (**g**) cellulose (waste cotton fabric).

The interactions among various components of the core-sheath fibers were investigated by FTIR analysis. As shown in Figure 2a–d, the core-sheath CS/CA–CNC fibers with various CS/CA ratios and CNC loading levels displayed analogous absorbance patterns, except the peaks corresponding to O-H stretching and N-H stretching vibrations [11,33]. Particularly, the broad peak at 3450 cm^−1^ shifted slightly to a lower wavenumber (3420 cm^−1^) as the mass proportion of CS rose, which disclosed the formation of intermolecular hydrogen bonds between hydroxyl groups of CA and amine groups of CS. In addition, the absorption bands at 1735 cm^−1^ and 1644 cm^−1^ were attributed to the carbonyl groups of CA and the C = O stretching of the acetyl groups in CS, respectively [34,35], but the absorption bands introduced by the addition of CNCs were implicit. It was due to the low contents of CNCs (up to 5 wt%) embedded in the core. The characteristic peaks of CA appeared in all the core-sheath CS/CA–CNC fibers (Figure 2a–d) at 1735 cm^−1^, 1370 cm^−1^, 1225 cm^−1^, and 1040 cm^−1^, corresponding to the stretching of carbonyl groups, methyl groups, C-O, and ether C-O-C of pyranose rings, respectively [36,37]. After the alkali hydrolysis, these four characteristic peaks were absent, but a strong absorption at 3330 cm^−1^ was observed (Figure 2e,f) [38]. Furthermore, the spectra of both CS/CL–CNC fibers were similar to that of cellulose (waste cotton fabric). These results revealed the successful deacetylation of CA. It was worth noting that the absorption peak corresponding to C = O stretching of the acetyl groups of CS (1644 cm^−1^) could still be observed in the spectra of CS/CL–CNC samples, which proved the retention of CS in the deacetylated fibers.

### 3.2. Core-Sheath CS/CA–CNC-Fiber Mechanical Properties

**Figure 3:**
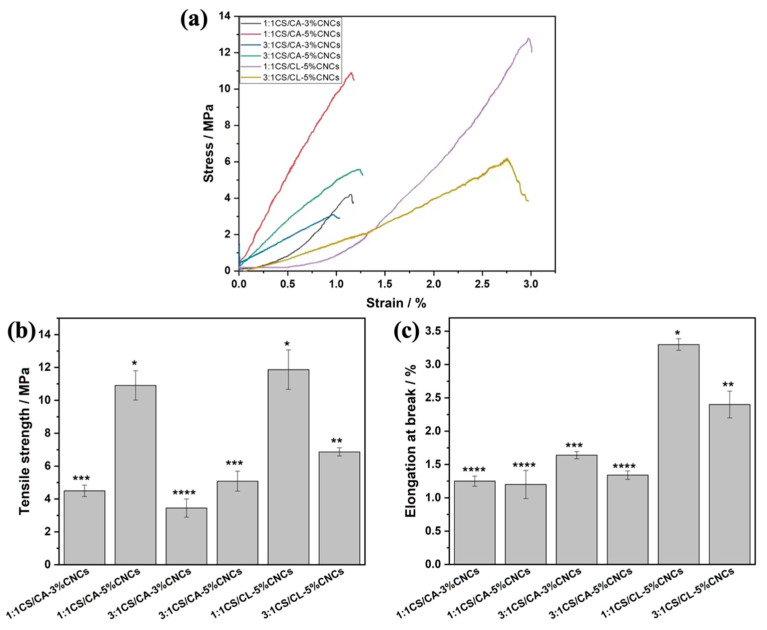
Typical stress–strain curves (**a**), tensile strength (**b**), and elongation at break (**c**) of various CS/CA–CNC fibers before and after deacetylation (different asterisks on the top of columns represent the significant difference (*p* < 0.05)).

The effects of CNCs and alkali hydrolysis on the mechanical properties of electrospun fibers were investigated. As shown in Figure 3, the content of CNCs in the core of CS/CA fibers had remarkable effects on their tensile strengths. Our former non-layered 1:1 and 3:1 CS/CA fibers had a tensile strength of 2.97 ± 0.59 and 0.22 ± 0.04 MPa [16], which were much lower than those of the core-sheath fibers containing CNCs. With further increase of CNC loading levels from 3 wt% to 5 wt%, both the 1:1CS/CA–CNC and 3:1CS/CA–CNC core-sheath fibers showed considerable improvements in the tensile strength, specifically from 4.50 ± 0.35 to 10.91 ± 0.89 MPa and 3.45 ± 0.55 to 5.08 ± 0.61 MPa, respectively. The distinguishable enhancement of the strength, on the one hand, was because the stress could be transferred and diverted from CS/CA fabrics to the rigid CNCs [28], and on the other hand, the interaction between CNCs and the fiber matrix also contributed to the resistance of loading forces [39]. Additionally, the dissimilarity of fibers in elongation at break was less noticeable. The incorporation of CNCs immobilized the CS/CA fibers to a certain extent, leading to an overall decreased elongation at break [24]. It was worth noting that the deacetylation of 1:1CS/CL–5%CNC and 3:1CS/CL–5%CNC fibers further reinforced the tensile strength to 11.87 ± 1.2 and 6.87 ± 0.25 MPa, respectively. It might be due to the conversion of CA into cellulose that facilitated the formation of hydrogen bonds within electrospun fibers [34].

### 3.3. Core-Sheath CS/CA–CNC-Fiber Adsorption Capacity

**Figure 4:**
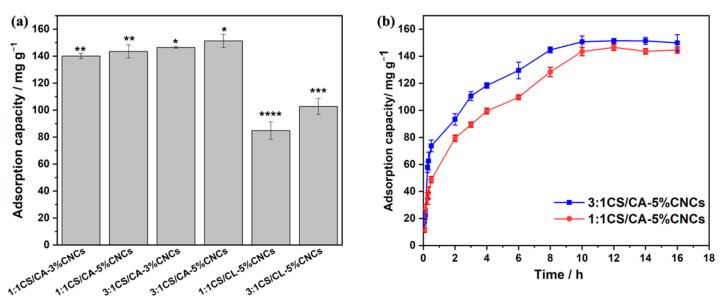
Maximum adsorption capacity of various core-sheath fibers (**a**), and effect of contact time on the adsorption capacity (**b**) (different asterisks on the top of columns represent the significant difference (*p* < 0.05)).

Figure 4a shows the experimental adsorption capacity of core-sheath CS/CA–CNC fibers under the optimal conditions (pH: 4.0, adsorbent dosage: 1.2 mg, volume of HA solution: 20 mL, initial concentration of HA solution: 30 ppm, shaking speed: 155 rpm, and temperature: 25 °C) [16]. The core-sheath 3:1CS/CA–CNC fibers exhibited obviously higher adsorption capacities towards HA than the 1:1CS/CA–CNC fibers. It was because the adsorption predominantly relied on the electrostatic attraction between the deprotonated carboxylic groups of HA and the protonated amino groups of CS [40], and more CS existed in the sheath layer of the 3:1CS/CA–CNC fibers. The adsorption capacity of the 3:1CS/CA–5%CNC fibers was 151.41 ± 1.76 mg/g, which had no significant difference compared to that of our former non-layered 3:1CS/CA fibers and was achieved by using less amount of CS in the electrospun fibers. This result demonstrated the importance of rational design in the fabrication of electrospun adsorbents. It was noted that the content of CNCs did not show any significant impact on the adsorption capacity. Therefore, the core-sheath 1:1CS/CA–5%CNC and 3:1CS/CA–5%CNC fibers that exhibited better mechanical properties were selected for the following study. As shown in Figure 4b, the adsorption of HA on both samples increased sharply and then gradually plateaued after 10 h. The initial fast increases in adsorption capacity implies the great availability of active sites, high specific surface area, and the porous structure of the electrospun fibers [17]. The alkali hydrolysis reduced the adsorption capacities to 84.8 and 102.69 mg/g for 1:1CS/CL–5%CNCs and 3:1CS/CL–5%CNCs, respectively. This result was attributed to the following two reasons: on the one hand, the interaction between hydrophobic moieties of HA and methyl groups of CA in the adsorbent contributed to the adsorption of HA, but these methyl groups were removed during the deacetylation of CA, and on the other hand, the hydroxyl groups of cellulose in the deacetylated fibers negatively affected the electrostatic interaction between the amine groups of CS and HA molecules [41]. However, these adsorption capacities toward HA were still acceptable compared to some adsorbents reported in previous studies [42,43,44,45,46]. For example, Wan Ngah et al. reported an adsorption capacity of 44.84 mg/g of HA (10 mg/L, 100 mL) using 0.05 g of CS–epichlorohydrin beads [45]. Similar to the current study, Thuyavan et al. also demonstrated a membrane type of adsorbent based on zirconia-embedded poly (ether sulfone), which exhibited an adsorption capacity of 50.5 mg/g towards HA and a tensile strength of up to 4.73 MPa [43].

## 4. Conclusions

This work demonstrated the importance of rational design in the fabrication of electrospun adsorbents and provided an effective method to recycle the waste materials through one-step co-axial electrospinning. All the co-axial CS/CA–CNC fibers exhibited smaller diameters and greater homogeneity compared to the non-layered ones. The presence of CNCs in the cores of fibers significantly improved the tensile strength, and a comparable maximum adsorption capacity to that of the non-layered fibers was achieved by the core-sheath design with lower total CS content. Additionally, the deacetylated CS/CL–CNC samples maintained the fibrous structure and showed an even higher tensile strength of 6.87 ± 0.25 MPa as well as an acceptable adsorption capacity of 82.69 ± 5.90 mg/g towards HA.

## Figures and Tables

**Table 1:** Various compositions and optimized electrospinning conditions of the core-sheath CS/CA–CNC fibers.

Samples	Core		Sheath		Electrospinning Conditions:
	CA Content (wt%)	CNC Content (wt% of CA Dry Weight)	Total Solid Content (wt%)	CS/CA Ratio	Applied Voltage (kV), Tip-to-Collector Distance (cm), Flow Rate (mL/h)
1:1CS/CA–5%CNCs	12	5	7	1:1	22, 11.5, 0.8 (sheath)-0.4 (core)
1:1CS/CA–3%CNCs	12	3	7	1:1	22, 11.5, 0.8 (sheath)-0.4 (core)
3:1CS/CA–5%CNCs	12	5	5	3:1	30, 9.5, 0.8 (sheath)-0.4 (core)
3:1CS/CA–3%CNCs	12	3	5	3:1	30, 9.5, 0.8 (sheath)-0.4 (core)

## Data Availability

The data presented in this study are available on request from the corresponding author.
